# Cooperative Chikungunya Virus Membrane Fusion and Its Substoichiometric Inhibition by CHK-152 Antibody

**DOI:** 10.3390/v14020270

**Published:** 2022-01-28

**Authors:** Jelle S. Blijleven, Ellen M. Bouma, Mareike K. S. van Duijl-Richter, Jolanda M. Smit, Antoine M. van Oijen

**Affiliations:** 1 Zernike Institute for Advanced Materials, University of Groningen, 9747 AG Groningen, The Netherlands; j.s.blijleven@rug.nl; 2 Department of Medical Microbiology and Infection Prevention, University Medical Center Groningen, University of Groningen, 9713 AV Groningen, The Netherlands; bouma92@gmail.com (E.M.B.); mareikeumcg@gmail.com (M.K.S.v.D.-R.); jolanda.smit@umcg.nl (J.M.S.); 3 Molecular Horizons, University of Wollongong, Wollongong, NSW 2522, Australia; 4 Illawarra Health and Medical Research Institute, Wollongong, NSW 2522, Australia

**Keywords:** chikungunya virus, single-particle, neutralizing antibody, membrane fusion

## Abstract

Chikungunya virus (CHIKV) presents a major burden on healthcare systems worldwide, but specific treatment remains unavailable. Attachment and fusion of CHIKV to the host cell membrane is mediated by the E1/E2 protein spikes. We used an in vitro single-particle fusion assay to study the effect of the potent, neutralizing antibody CHK-152 on CHIKV binding and fusion. We find that CHK-152 shields the virions, inhibiting interaction with the target membrane and inhibiting fusion. The analysis of the ratio of bound antibodies to epitopes implied that CHIKV fusion is a highly cooperative process. Further, dissociation of the antibody at lower pH results in a finely balanced kinetic competition between inhibition and fusion, suggesting a window of opportunity for the spike proteins to act and mediate fusion, even in the presence of the antibody.

## 1. Introduction

The chikungunya virus (CHIKV; Alphavirus genus, Togaviridae family) is a human arthropod-borne virus causing chikungunya fever and potentially long-lasting effects, such as joint pain. It has recently greatly expanded its geographic range to encompass most tropical-to-temperate regions of the world [1] and is likely to spread further, due to geographic expansion of the mosquito vectors that transmit the virus [2,3,4]. No preventive medicine or specific antiviral treatment is available to counter CHIKV infection.

Alphaviruses are enveloped viruses in which the lipid bilayer is derived from the host plasma membrane [5]. The membrane encapsulates the protein capsid in which the viral genome resides. Two viral proteins, E1 and E2, are anchored into the membrane and arranged in trimers of E1/E2 heterodimers called spikes. The spikes cover the surface in an icosahedral lattice with triangulation *t* = 4, giving rise to 80 spikes, or 240 copies of the E1/E2 heterodimers in total [6]. The E2 protein facilitates alphavirus binding to cellular receptors [7,8], and both the E1 and E2 proteins play an important role in the process of membrane fusion.

A critical step in the reproductive cycle of enveloped viruses involves the merger of the viral membrane with the host cellular membrane to deliver the genome to the host cell to start a new cycle of viral replication (reviewed by Harrison [9]). However, membrane fusion does not occur spontaneously on biological timescales, due to high kinetic barriers between the intermediates [10]. Enveloped viruses, therefore, have envelope proteins that catalyze membrane fusion (reviewed by Kielian [11]), to deliver the viral genome at the right time to the right place in the host cell. Upon attachment of CHIKV to the cell, the virion is taken up into an endosomal compartment, mainly by clathrin-mediated endocytosis [12]. Membrane fusion is initiated at the mildly acidic pH of the early endosome [13,14], triggering the E1/E2 heterodimers to dissociate [6,15]. The E1 proteins subsequently insert themselves into the endosomal membrane and trimerize to form the functional units of fusion [16,17]. Multiple trimers are thought to be necessary to concertedly bring both membranes together [14,18,19], first leading to a hemifused intermediate where the proximal leaflets have merged, and finally opening a pore to deliver the viral genome into the cellular cytosol.

There is currently no vaccine or treatment available against CHIKV, but several promising antibodies have been isolated and were shown to prevent CHIKV infection [20]. A potent antibody is CHK-152, which was found to protect against CHIKV infection in mouse and nonhuman primate models [21,22]. Mutational and cryo-EM reconstruction studies showed that it binds to the acid-sensitive region of E2. This region becomes disordered at low pH, thereby facilitating exposure of the E1 fusion loop [6,23,24].

In this study, we found that CHK-152 strongly interferes with CHIKV membrane interactions, both at neutral and low pH. Additionally, in a single-particle fluorescence microscopy assay, the fusion of particles that were already docked to the membrane was blocked and slowed down. At pH 6.1 and substoichiometric antibody binding, fusion was efficiently inhibited. This effect was diminished at pH 5 and 4.7, as at these pH values, CHK-152 was found to dissociate from the virus particles. We explain the results in a model of CHIKV fusion as being mediated by multiple E1 trimers formed from CHK-152-free spikes. The stoichiometry of antibody binding implies a cooperative fusion mechanism, where three to five neighboring E1 trimers are required to mediate membrane fusion.

## 2. Materials and Methods

The CHIKV strain LR2006-OPY1 was a kind gift from Dr. Andres Merits. The antibody CHK-152 was a kind gift from Dr. Michael Diamond. Assays were performed at 37 °C, except the single-particle assay, which was performed at room temperature (approximately 22 °C). The corresponding change in the rate of fusion was determined in the liposomal fusion assay, described below (Figure A2). Throughout this section, we refer to (hemi)fusion as fusion, as the assays used do not distinguish content mixing from lipid mixing. The appendix contains details of the hypothesis testing (Table A1) and fitting (Table A2).

Virus—radiolabeled. A confluent layer of BHK-21 cells was infected at an MOI of 10. The virus inoculum was removed after 2.5 h incubation and following a 1.5 h starvation, 200 µCi (7.4 MBq) [35S]-methionine/L-[35S] cysteine using EasyTag ™ EXPRESS35S Protein Labeling Mix (PerkinElmer, Groningen, The Netherlands) was added to the medium. Supernatant was harvested 20 hpi (hours postinfection) and layered on top of a two-step sucrose gradient (20%/50% *w*/*v* in HNE) and centrifuged for 2 h at 154,000× *g* at 4 °C in a SW41 rotor (Beckman Coulter, Woerden, The Netherlands) to clear it from the cell debris. Radioactive virus was collected at the 20%/50% sucrose interface and radioactivity was counted by liquid scintillation analysis. The fractions were pooled based on radioactivity counts. The infectivity of the virus preparation was determined by standard plaque assay on Vero–WHO cells and by qRT-PCR to determine the number of genome-containing particles, as described previously [14].

Virus—fluorescently labeled and inactivated. Virus stocks were prepared as described previously [14]. Briefly, CHIKV seed stocks were prepared by the infection of Vero--WHO cells at a multiplicity of infection (MOI) of 0.01. Pyrene-labeled virus was produced in BHK-21 cells cultured beforehand in the presence of 15 μg/mL 1-pyrenehexadecanoic acid (Thermo Fisher Scientific, Waltham, MA, USA). The supernatant was harvested at 48 hpi, cleared from cell debris by low-speed centrifugation, purified by ultra-centrifugation, and frozen in liquid nitrogen. Before freezing, the virus was UV-inactivated as the single-particle fusion assay was performed outside the BSL-3 facility [14]. To produce octadecyl rhodamine B chloride (R18; Thermo Fisher Scientific)-labeled virions, 7.2 × 10^12^ particles of purified and inactivated CHIKV were diluted in PBS (10 mM phosphate, 140 mM NaCl, 0.2 mM EDTA) and 0.3 µL of 0.2 mM R18 dissolved in DMSO was added to a final concentration of 1 μM. Subsequently, the virus solution was kept on ice for 1 h. A gel-filtration column (PD-10 desalting column; GE Healthcare, Hoevelaken, The Netherlands) was used to separate the virus from unincorporated dye. The most concentrated fractions were combined and used in the experiment.

Liposomes. Liposomes were prepared as described previously [14,25]. For the non-single-particle assays, the liposomes consisted of sphingomyelin from porcine brain, transphosphatidylated L-α-phosphatidylethanolamine (PE) from chicken egg, L-α-phosphatidylcholine (PC), and cholesterol from ovine wool. The lipids were mixed in a molar ratio of 1:1:1:1.5. The liposomes were prepared by freeze–thaw extrusion and extruded through a polycarbonate membrane with 200 nm pore. All lipids and the polycarbonate membrane were purchased from Avanti Polar Lipids (Alabaster, AL, USA). Lipids and the phospholipid-to-cholesterol ratio were chosen to approximate the lipid composition within the endosomal compartment [26,27]. For the single-particle assay, liposomes (200 nm) were also prepared by freeze–thaw extrusion. Liposomes consisted of 1:1:1:1.5:2 × 10^−5^ ratio of 1,2-dioleoyl-sn-glycero-3-phosphocholine (DOPC), 1,2-dioleoyl-sn-glycero-3-phosphoethanolamine (DOPE), porcine brain sphingomyelin (SPM), ovine wool cholesterol, and 1,2-dioleoyl-sn-glycero-3-phosphoethanolamine-N-(biotinyl) (Biotin-PE).

Trypsin cleavage of CHIKV structural proteins at neutral pH. The [35S]-methionine/L-[35S] cysteine-labeled CHIKV was incubated for 10 min at 37 °C with CHK-152 in HNE in the appropriate ratio. In the final volume for the tested conditions were 0.63 nM CHK-152 in an approximate ratio of 13 to virions, and 10 nM CHK-152 in ratio of 210 to virions. Thereafter, liposomes were added at a final concentration of 200 µM at 37 °C in a total volume of 133 µL HNE buffer (5 mM HEPES, 150 mM NaCl, 0.1 mM EDTA) and kept for 60 s at pH 7.4. The mixture was digested with N-tosyl-L-phenylalanyl chloromethyl ketone (TPCK)-treated trypsin (Sigma-Aldrich, St. Louis, MO, USA) at a concentration of 200 µg/mL in the presence of 1% Triton X-100. After 1 h at 37 °C, the samples were subjected to SDS-PAGE analysis.

Trypsin cleavage of E1 homotrimer at low pH. The [35S]-methionine/L-[35S] cysteine-labeled CHIKV was prior opsonized with CHK-152: 20 nM CHK-152 in a ratio of 335 to virions. This was then mixed with 200 µM liposomes at 37 °C in a total volume of 133 µL HNE buffer (5 mM HEPES, 150 mM NaCl, 0.1 mM EDTA). After 60 s of incubation, the pH was lowered to pH 5.1 by the addition of 7 μL of a pretitrated buffer (0.1 MES, 0.2 M acetic acid, NaOH to achieve the desired pH). After 60 s, the mixture was neutralized to pH 8.0 by the addition of 3 µL of pretitrated NaOH solution. The samples were incubated in 0.25% β-mercaptoethanol (β-ME) for 30 min and subsequently digested with TPCK-treated trypsin (Sigma) at a concentration of 200 µg/mL in the presence of 1% Triton X-100. The samples were then subjected to SDS-PAGE analysis.

SDS-PAGE analysis. The samples were solubilized with 4× SDS sample buffer (Merck–Millipore, Darmstadt, Germany) and analyzed with SDS-PAGE on 10% Mini-PROTEAN ^®^ TGX ™ Precast Protein Gels (Bio-Rad, Hercules, CA, USA). The gels were fixed in 1 M sodium salicylate for 30 min and dried. Viral protein bands were visualized in a Cyclone Plus Phosphor Imager (PerkinElmer) and radiographs were further analyzed using ImageQuant.

Cofloatation assay. The influence of antibody binding of CHIKV on low-pH-induced liposome-binding was assessed using a cofloatation assay described previously for SFV and SINV [16,25,28]. Briefly, 0.75 μM viral phospholipid of [35S]-methionine/L-[35S]-cysteine-labeled CHIKV particles was mixed with 200 μM liposomes in HNE buffer. The mixture was acidified by adding a pretitrated amount of low pH buffer (0.1 M MES, 0.2 M acetic acid, NaOH to achieve the desired pH). At 60 s after acidification, the mixture was neutralized to pH 8.0 by NaOH and placed on ice. A volume of 100 μL of this fusion reaction was added to 1.4 mL of 50% sucrose in HNE (*w*/*v*). A sucrose density gradient was prepared consisting of 60% sucrose in HNE, followed by 50% sucrose in HNE including the fusion mixture, and 20% sucrose in HNE and 5% sucrose in HNE on top. Gradients were centrifuged in a SW55 Ti rotor (Beckman Coulter) for 2 h at 150,000× *g*. The gradient was fractionated in ten parts, and radioactivity in each fraction was determined by liquid scintillation analysis. The relative radioactivity in the top four fractions compared to total radioactivity in the gradient was taken as the measure for CHIKV that were bound to liposomes. For antibody inhibition, [35S]-methionine/L-[35S]-cysteine-labeled CHIKV was incubated for 10 min at 37 °C with 10 nM of CHK-152 in HNE before proceeding with a fusion measurement as described above. As an antibody control, Mouse IgG2A Isotype Control catalog number MAB0031 was used.

Single-particle fusion—assay and microscopy. Experiments were performed at room temperature as reported previously [14,29]. Glass microscope coverslips (24 mm × 50 mm, No. 1.5; Marienfeld brand, VWR, Amsterdam, The Netherlands) were cleaned by 30 min sonication in acetone and ethanol, followed by 10 min sonication with 1 M potassium hydroxide and finally 30 min cleaning in an oxygen plasma cleaner. The last step was performed on the day of measurement. Polydimethylsiloxane (PDMS) flow cells with a channel cross-section of 0.1 mm^2^ were prepared as previously [29]. Imaging was performed with near-total internal reflection fluorescence microscopy (TIRF-M), using an inverted microscope (IX-71, Olympus, Leiderdorp, The Netherlands) and a high numerical aperture with an oil-immersion objective (NA 1.45, 60×; Olympus). Liposomes were flushed into the flow cell and a planar lipid bilayer was allowed to form for >50 min. Virions were docked nonspecifically to the lipid bilayer for 3 min at 50 μL/min. Fluorescein-labelled streptavidin (Thermo Fisher Scientific) was introduced into the flow cell at 0.2 μg/mL for 5 min at 10 μL/min, as a pH drop proxy. Then, PBS with 2 mM Trolox ((±)-6-Hydroxy-2,5,7,8-tetramethylchromane-2-carboxylic acid, Sigma-Aldrich) was flown in for 2 min at 100 μL/min to remove unbound virions and fluorescein. The presence of Trolox prevented laser-intensity-dependent fusion inactivation, presumably by reducing oxidative damage from the fluorescent dye. The aqueous environment was acidified by flowing in citric acid buffer (10 mM, 140 mM NaCl, 0.2 mM EDTA) of pH 5.1 at 600 μL/min. The fluorophores were excited using 488 nm and 561 nm lasers (Sapphire, Coherent Inc., Santa Clara, CA, USA). Viral membrane fluorescence (red) and fluorescein pH drop fluorescence (green) were projected onto different halves of an EM-CCD camera (C9100-13, Hamamatsu, Iwata-shi, Shizuoka-ken, Japan). Exposure time was 300 ms. Opsonization was performed for 15 min at 37 °C with appropriate concentrations of the antibody and 10× diluted labeled virus, in the final volume.

Antibody labeling and characterization. CHK-152 was labeled with AlexaFluor488 TFP-ester (Thermo Fisher Scientific) per the manufacturer’s guidance. UV-VIS spectroscopy indicated a labeling ratio of 1.5 dye/CHK-152; tandem MALDI mass spectrometry was consistent with this (Figure A5). MALDI was performed in 150 mM ammonium acetate after dialysis. From the labeling ratio, we estimated the fraction of unlabeled (i.e., not visualized) CHK-152 at 0.22, by assuming a Poissonian labeling distribution. To determine single CHK-152 intensity, labeled CHK-152 was flown in at a roughly picomolar concentration into a clean flow cell, as described above. The imaging conditions and buffers were the same as for the virions (i.e., pH 5.1, unless noted otherwise). Single CHK-152 intensity was determined in a 7 × 7-pixel region, to be 36 ± 2 A.U. per CHK-152 (Figure A6a), corrected for background and laser intensity. Antibody fluorescence intensity was independent of pH (Figure A6b). At the timescale of the experiments (2 to 4 min) no bleaching of dye was observed.

Single-particle fusion—analysis. The ImageJ program in the FIJI project, together with home-written software in MATLAB, were used to extract the fluorescence signals, essentially as described previously [14,29,30,31]. In brief, the fluorescein pH-drop signal was integrated over the entire field of view, and the *t* = 0 of the experiment, defined as the time point, was where only 8% of a fitted sigmoidal function remained. The particles’ fusion events and times were manually detected by inspecting the virion R18 intensity traces, together with the movie. CHK-152 fluorescence traces were extracted in a 7 × 7-pixel region, corrected for background, laser intensity, and laser illumination profile, and divided by the intensity per CHK-152 and dark fraction, as determined above, to yield the number of CHK-152 bound. As we detected virion aggregation, presumably by antibody crosslinking, in both an increased R18 intensity distribution and a bimodal CHK-152 distribution (Figure A4a,b), we only analyzed virions with up to 90 CHK-152 bound. These fell within a normally distributed portion of the population (Figure A4b), in contrast with the lognormally distributed tail, and comprised 75% of the total number of virions observed.

Simulations. To determine the average number of unbound spikes, the following formula was applied, with $p_{unboundSpike}$ as the probability that a spike (consisting of three monomers) is not bound on any epitope, $p_{unboundMonomer}$ as the probability that a monomer has the epitope unbound, $n_{boundEpitopes}$ as the number of epitopes bound in total, and $n_{epitopes}$ as the total number of epitopes (for CHIKV, 240 epitopes).

$$p_{unboundSpike}={\left(p_{unboundMonomer}\right)}^{3}={\left(1-n_{boundEpitopes}/n_{epitopes}\right)}^{3}$$

For monovalent antibody binding, *n_boundEpitopes_* is equal to the number of bound antibodies. For the theoretically maximum bivalent binding, where each antibody binds two epitopes exactly, the *n_boundEpitopes_* is two times that amount.

Numerical simulations were performed in MATLAB. A grid of spikes was defined per Figure A8b, where patch sizes from 12 to 40 (half a virion) were considered. Each spike contained 3 epitopes, and a specified number of inhibitors was bound randomly across all epitopes. This number of antibodies, or the related quantity of epitope occupancy (number of antibodies divided by number of epitopes), was varied. Statistics were obtained for 10,000 virions. The number of unbound spikes within the contact patch was counted separately, and in the context of the defined 5- and 6-rings in Figure A8b. The extent of fusion was defined as the fraction of virions that had at minimum one 5- or 6-ring with N_H_ unbound spikes, as detailed in the main text. To facilitate comparison with the numerical model, the data were scaled to take into account dissociation. Effective number of CHK-152 bound: the average number of CHK-152 over nonfusing virions was averaged over time weighted by the number of unfused virions. This is, therefore, a measure for the average number of CHK-152 a fusing virion had bound, during the time it took to fuse. Relative extent of fusion: the extent of fusion in the presence of CHK-152 was divided by the extent of fusion without the antibody. The relative extent of fusion, therefore, is corrected for virions that were never able to fuse, and for the pH variability of the fusion extent.

## 3. Results

To delineate the different mechanistic effects of the CHK-152 antibody on the fusion process, we set out to separately characterize membrane binding and fusion. By using a combination of binding assays, we studied the effect of CHK-152 on membrane interactions. These experiments were followed by a single-particle assay with pre-docked particles to directly investigate the effect of CHK-152 on membrane fusion, and to determine the stoichiometry of neutralization.

### 3.1. CHK-152 Shields Virions, Thereby Preventing Neutral-pH Membrane Interaction

**Figure 1:**
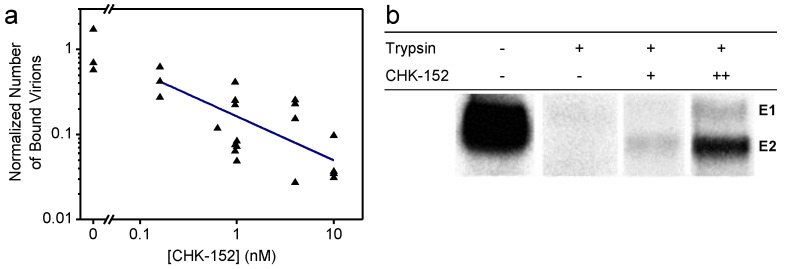
Shielding of virions by CHK-152 at neutral pH; (**a**) Inhibition of nonspecific binding to a planar membrane. Fluorescently labeled CHIK virions were incubated with CHK-152, flown into a flow cell, and docked to a planar membrane (see text). The number of particles binding to the membrane after rinsing the channel was counted and normalized to the mean number of particles in the absence of the antibody. Single trials shown on log–log scale (*n* = 25); blue line indicates a power-law fit with power coefficient −0.5 ± 0.2; (**b**) shielding of surface proteins from enzymatic cleavage. The [35S]-methionine/L-[35S] cysteine-labeled CHIKV was incubated with CHK-152 and mixed with liposomes at neutral pH. The mixture was trypsinized for 1 h and subjected to SDS-PAGE analysis. CHK-152 concentration in final volume: +, 0.63 nM CHK-152 in estimated ratio of 13 to virions; ++, 10 nM CHK-152 in ratio of 210 to virions. Triplicate trials included in Figure A13.

First, we wanted to determine the effect of CHK-152 on nonspecific membrane interaction at a neutral pH. A planar, lipid membrane was formed by using a flow cell constructed on top of a hydrophilic microscope coverslip and introducing liposomes [32]. The receptor-free bilayer incorporated DOPC, DOPE, sphingomyelin, and cholesterol, the latter two lipids being stimulating and required factors for fusion [14,33,34,35]. CHIKV particles were UV inactivated to render them noninfectious and were labeled with the lipophilic dye R18. After labeling, they were incubated with varying concentrations of the CHK-152 antibody and flown into the flow cell to dock to the membrane. After rinsing with buffer, the number of particles sticking to the bilayer was quantified by single-particle fluorescence microscopy (more detail below and in Methods). The particle counts normalized to the same conditions, but in the absence of CHK-152 are shown in Figure 1a on double-log scale.

We found that nonspecific binding reduced with the increasing concentration of CHK-152 during the preincubation phase, as indicated by the fit of a power function (linear on a log–log scale). Interestingly, we also found that CHK-152 shields the E2 surface glycoprotein from enzymatic cleavage by trypsin (Figure 1b). Radiolabeled CHIKV was mixed with liposomes at neutral pH and subjected to trypsin digestion and SDS-PAGE analysis. Trypsin completely digested the E1 and E2 proteins, while preincubation with increasing concentrations of CHK-152 protected the E2 protein from trypsin digestion, indicating that E2 proteins were shielded against enzymatic cleavage. Collectively, these results suggest that the CHIKV membrane interaction at neutral pH is reduced due to steric hindrance caused by the CHK-152 antibody.

### 3.2. CHK-152 Blocks Interaction with Target Membranes at Low pH

At a low pH, the viral fusion proteins undergo conformational changes to support membrane fusion. Antibodies have been described that prevent the conformational changes that are required for membrane fusion or that freeze virus particles in an intermediate stage [36,37,38,39,40]. We described previously that CHK-152 abolishes membrane fusion activity at a high antibody concentration in a liposomal fusion assay [21]. There, we investigated the effect of CHK-152 on CHIKV fusion, and revealed that both the extent as well as the rate of fusion decreases with increasing antibody concentrations [21,41]. At 10 nM CHK-152, membrane fusion was almost completely abolished.

**Figure 2:**
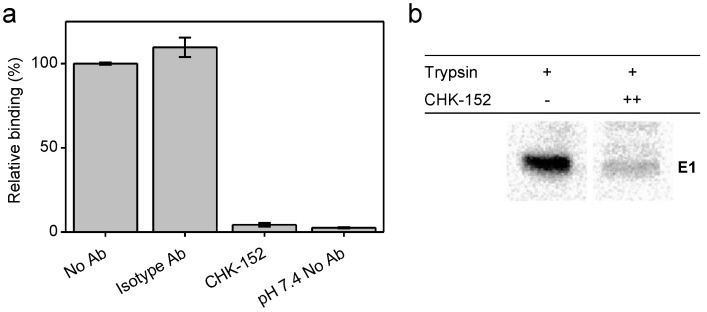
CHK-152 inhibition of target membrane interaction at low pH; (**a**) inhibition of E1-liposome interaction at low pH. A fusion experiment was performed, adding radiolabeled CHIKV that was preincubated, without or with 10 nM of isotype control or CHK-152 antibodies to liposomes, and acidifying the mixture to pH 5.1. After 1 min, the sample was neutralized and added to the bottom of a sucrose gradient and centrifuged. The relative radioactivity in the top fractions, therefore, cofloating with the liposomes, was determined in triplicate and is plotted as mean ± sem; (**b**) inhibition of formation of trypsin-resistant E1 trimer. Radiolabeled CHIKV was incubated with or without CHK-152 for 10 min at 37 °C, added to liposomes and acidified to pH 5.1. After 1 min, the sample was neutralized to pH 8.0. The sample was incubated with 0.25% β-ME for 30 min at 37 °C, trypsinized for 1 h, and subjected to SDS-PAGE analysis. CHK-152 concentration at incubation: ++, 20 nM CHK-152 in ratio of 335 to virions. Triplicate trials included in Figure A14.

To further dissect the role of CHK-152 on membrane fusion, we here determined the low-pH-dependent binding properties of the virus to liposomes in the presence or absence of CHK-152, by the use of a liposomal cofloatation assay (Figure 2a).

Radiolabeled CHIKV preincubated with 10 nM CHK-152 was added to liposomes, after which the mixture was acidified to pH 5.1 for 1 min and back-neutralized to pH 8.0. A sucrose density column was formed from a layer of 60% (*w*/*v*) sucrose, then the sample mixed with 50% sucrose, and on top of that, 20% and 5% layers. Upon ultracentrifugation, liposome-bound virus particles are at the 5–20%-layer interface, whereas unbound particles remain within the 50% sucrose layer. The radioactivity counts were determined, providing a measure of virus cofloating with, and therefore bound to, the liposomes. In the absence of antibodies, on average, 55% binding was observed that was set to 100%. Comparable virus–liposome binding was observed in the presence of an isotype antibody. Importantly, however, virus–liposome binding was completely abolished in the presence of CHK-152 antibodies. This observation suggests that CHK-152 prevents stable interaction of E1 to liposomes, and as a consequence, no membrane fusion is observed.

To investigate whether CHK-152 indeed blocks the low-pH-induced conformational changes that are required for membrane fusion, we assessed the formation of a trypsin-resistant form of E1 under low-pH conditions (Figure 2b). It is known that the E1 homotrimer of alphaviruses that is formed upon low-pH treatment is resistant to trypsin digestion [42]. The trypsin-resistant E1-trimer dissociates into monomers when boiled in SDS sample buffer, and can be detected with SDS-PAGE analysis. CHK-152-opsonized, radiolabeled CHIKV was incubated with liposomes at pH 5.1, as described for the liposome-binding assay (also see Methods). After back-neutralization to pH 8.0, the acidified liposome-CHIKV mixture was incubated with the reducing agent β mercaptoethanol for 30 min in order to make the proteins more accessible to trypsin cleavage. The sample was then subjected to trypsin digestion. As expected, in the absence of CHK-152, a clear trypsin-resistant E1-band was seen. In presence of 20 nM CHK-152, however, the formation of the trypsin-resistant form of E1 was markedly reduced. Collectively, these observations suggest that high concentrations of CHK-152 either freeze the particle in the original state or interfere with an early step in the membrane fusion process, i.e., at a step prior to stable interaction of E1 with the target membrane.

### 3.3. The Single-Particle Assay

We established that CHK-152 blocks efficient membrane interaction, both at neutral and low pH, at high antibody concentrations. At lower antibody concentrations, however, CHIKV was able to bind to planar bilayers (Figure 1a), and we aimed to elucidate whether CHK-152 is able to directly interfere with membrane fusion at these conditions, and if so, to determine the stoichiometry of CHK-152-mediated neutralization of membrane fusion. To this end, we employed a single-particle assay with fluorescently tagged CHK-152, enabling the counting of CHK-152 bound to the individual viral particles. The single-particle assay relies on a controlled in vitro environment that enables synchronized acidification to initiate fusion, and uses fluorescent tags to correlate the rate and extent of fusion to antibody binding.

**Figure 3:**
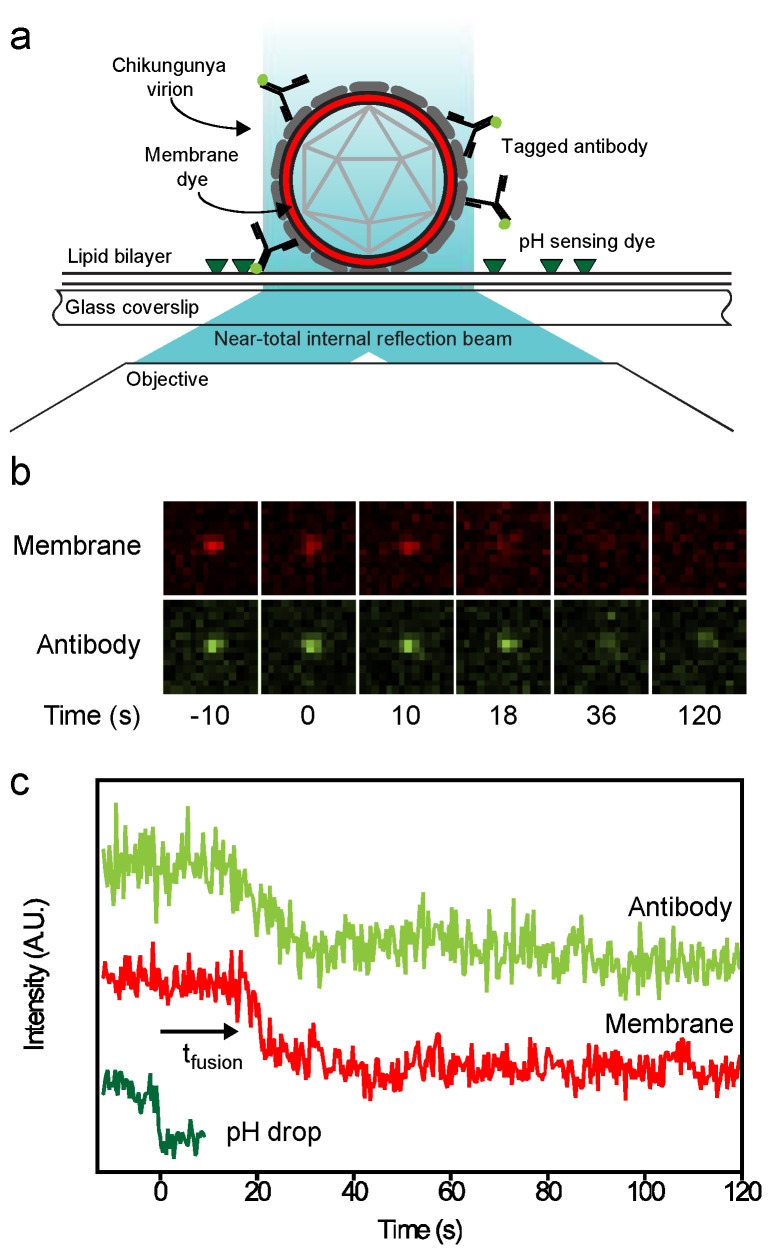
Single-particle assay; (**a**) in a flow channel, a lipid bilayer was formed on a cover glass. Viruses were labeled with lipophilic dye R18 and docked nonspecifically. A pH-sensitive dye attached to the membrane reported on pH change in the channel. Antibodies were detected and counted through a fluorescent tag. Fluorescence was excited by laser beams leaving the coverslip at a small angle. Fluorescence was split and projected onto different halves of a camera, allowing colocalization of the viral membrane and antibody spots; (**b**) examples of observed fluorescence (membrane and antibody) of the same virus particle. Hemifusion could be seen around 16 s after acidification as escape of the membrane dye into the target bilayer. Loss of antibody intensity was also observed; (**c**) intensity information collected from the virus particle in panel b. Top trace shows the loss of antibodies over time after acidification. Middle trace shows the membrane intensity signal. The lower trace shows the disappearance of fluorescence of the fluorescein pH probe, defining the start of the experiment. The time to hemifusion, defined as the onset of signal dissipation, is indicated as t_fusion_.

The essentials of the single-particle assay are illustrated in Figure 3. The features were similar to those described previously [14,29]. The basis is an in vitro flow cell system that allows rapid acidification of virions that are predocked onto a planar lipid bilayer (Figure 3a), monitoring, at the same time for every particle, the occurrence of hemifusion and the number of antibodies present. As described above, a planar lipid bilayer was formed on a hydrophilic coverslip in a flow cell. A biotinylated lipid provided an anchor for fluorescein-labeled streptavidin to report on the change in local pH. CHIKV particles were membrane-labeled with the lipophilic dye R18, incubated at 37 °C with or without the antibody, and flown into the flow cell to dock nonspecifically to the bilayer. After acidification, hemifusion was observed as the escape of R18 from the viral membrane into the target bilayer (Figure 3b), and the time from pH drop to hemifusion was determined.

### 3.4. CHK-152 Blocks and Slows down Fusion of Pre-Docked Virions in a pH-Dependent Manner

**Figure 4:**
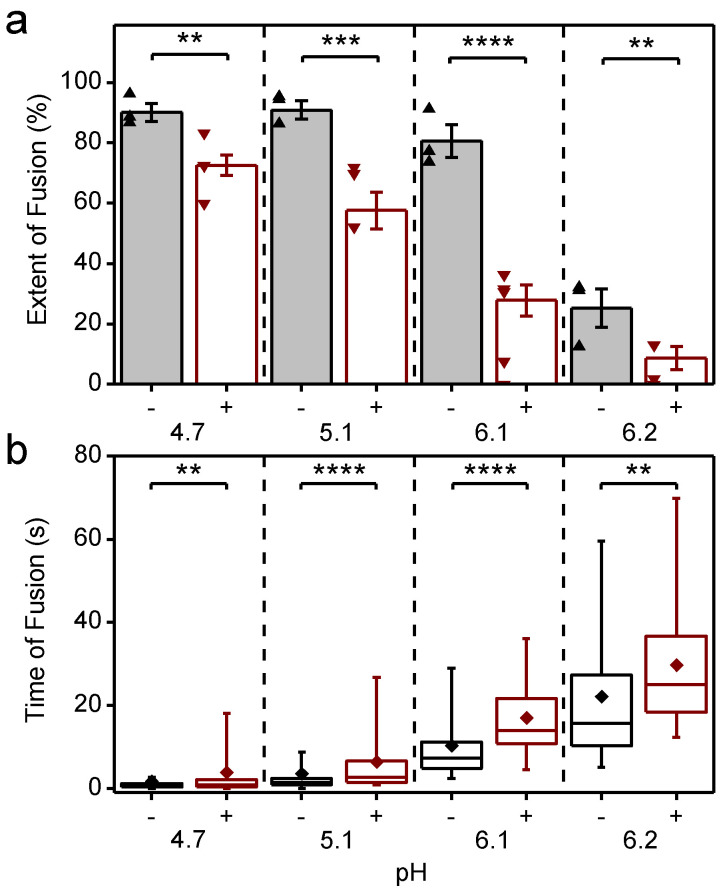
Inhibition and slow-down of CHIKV fusion by CHK-152, in a pH-dependent manner; (**a**) virions pre-docked to the planar bilayer were acidified to the pH-point indicated below the *x*-axis, either with (+) or without (−) preincubation with CHK-152. The extent of fusion, the fraction of the population undergoing fusion, is shown. Mean ± sem shown together with single experiments (triangles): black/-, without CHK-152, red/+, with preincubation of 0.63 nM CHK-152, resulting in 52 ± 3 CHK-152 bound (see text). Significances determined by weighted *t*-test; (**b**) time of hemifusion of single particles with the same color coding of conditions as panel a. Means, diamonds; box plots, 5%-Q1-median-Q3-95% intervals. Significance of difference of medians determined by Wilcoxon rank–sum test. Obtained *p*-values (Table A1) **: *p* < 0.01, ***: *p* < 0.001, ****: *p* < 0.0001. pH 6.1 and 6.2 lay at the threshold of fusion (Figure A1). Fusion was studied at room temperature as the rate of fusion scaled in an Arrhenius-like fashion over the range 37 °C to room temperature, as determined with the liposomal fusion assay (Figure A2). Figure A3 details the CHK-152 numbers bound and shows no correlation between the starting number of CHK-152 and the fate of fusion. There was some antibody-induced virion aggregation and, therefore, virions with high antibody counts were filtered out (Figure A4 and Methods). Videos S1–S8 show representative timelapses of each condition.

**Figure A1:**
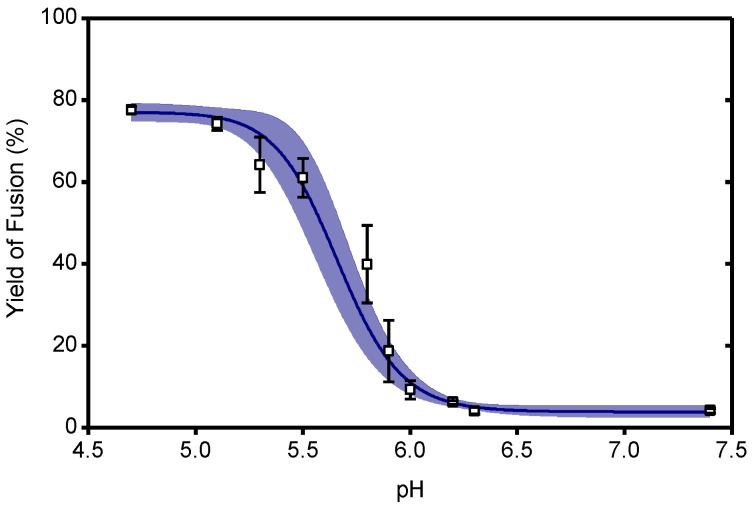
pH-dependence of the extent of fusion in a liposomal fusion assay for the CHIKV LR2006 OPY1 strain. Pyrene-labeled viruses were mixed with liposomes and acidified to the indicated pH at 37 °C. The yield of fusion was determined as the amount of fluorescence detected at 60 s relative to full dilution of the pyrene probe by detergent (see Methods). A logistic curve was fitted to the data, 95% confidence intervals indicated. Supplement to Figure 4.

**Figure A2:**
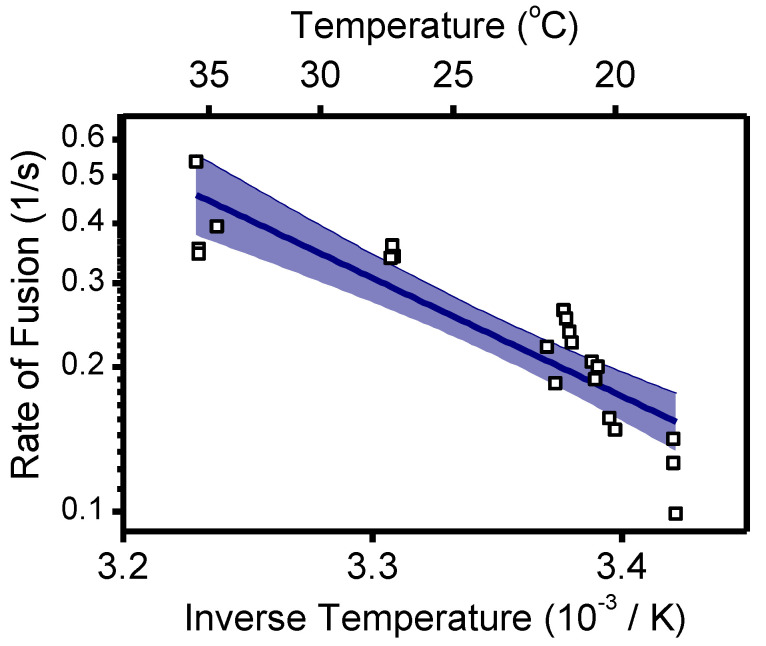
Arrhenius plot of the rate of fusion in a bulk liposomal fusion assay versus the inverse temperature. Pyrene-labeled viruses were mixed with liposomes and acidified to pH 5.1 at the specified temperature. The rate of fusion was determined as the inverse of the time to reach 50% of the maximum fusion extent (see Methods). *n* = 21 trials. Linear fit with 95% confidence interval indicated. Supplement to Figure 4.

To correlate the effect of CHK-152 to different fusion conditions, we determined the fusion extent and time to fusion at pH 6.2, 6.1, 5.1, and 4.7. The latter two pH points lie in the optimal regime of fusion, and the first two around the threshold of fusion activation (see Figure A1 and [14]). Measurements at pH 6.2 and 6.1 are in the pH range of early endosomes from which CHIKV particles have been described to fuse [13]. We studied fusion at room temperature; the rate of fusion scaled in an Arrhenius-like fashion over the range 37 °C to room temperature, as determined with the liposomal fusion assay described above (Figure A2). The extent of fusion, the fraction of the particle population that undergoes hemifusion within 2 min after acidification, is shown in Figure 4a.

Fusion was highly efficient, with experiments showing up to 96% extent of fusion. As previously observed for the S27 strain [14], the LR2006-OPY1 strain exhibited a sharp pH threshold between pH 6.2 and 6.1, with the extent of fusion reduced by half over a pH difference of 0.1. The time to hemifusion of single particles is shown in Figure 4b, and shows that the time to fusion is longer with a higher pH.

**Figure A3:**
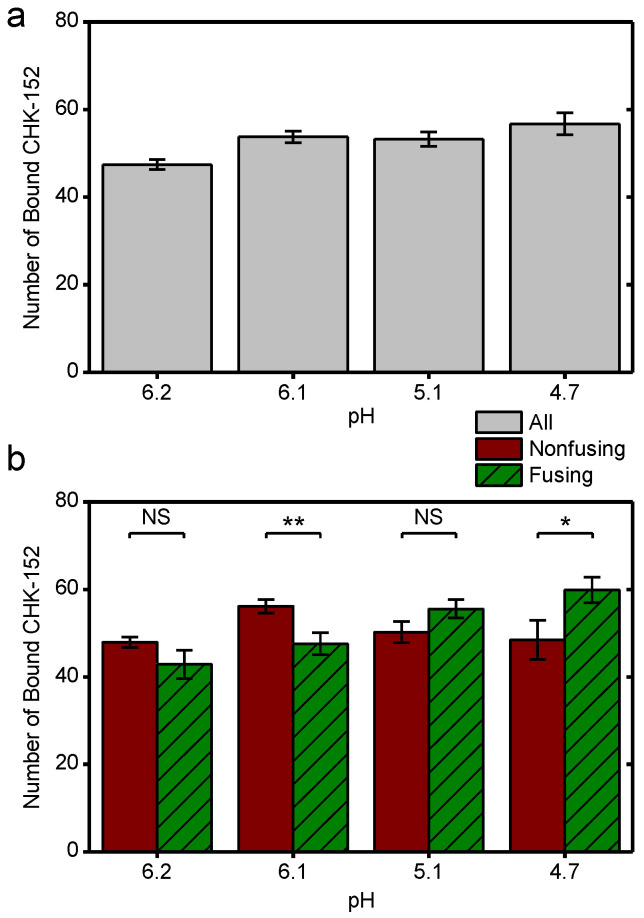
Number of CHK-152 bound at the start of the experiment per pH condition. As described in the Methods, the number of CHK-152 per virion was determined in the single-particle assay at *t* = 0. These numbers are here shown as means for different subsets of all virions. (**a**) All virions: number of CHK-152 bound at start for all particles taken together. (**b**) The data as in panel a split into the subpopulations of viruses that fuse and those that do not. Significances determined by *t*-test. * *p* < 0.05, ** *p* < 0.01. Supplement to Figure 4.

**Figure A4:**
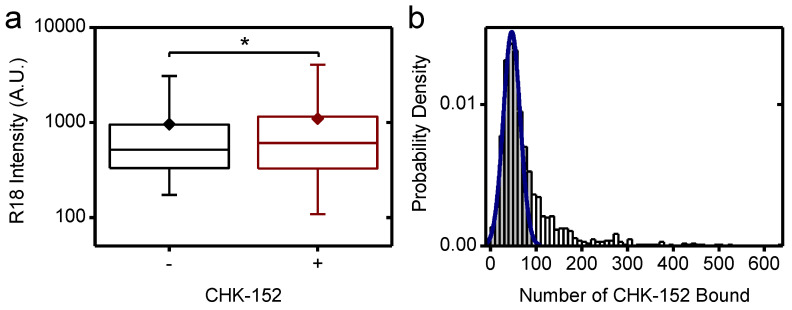
CHK-152-induced virion aggregation. (**a**) For virions docked to the planar bilayer at pH 7.4, the R18 intensity was determined and is shown on a log scale. Conditions: −, without CHK-152 and +, with CHK-152 preincubation. Significance determined by *t*-test on the means, *n*_−_ = 2149 and *n*_+_ = 1042 virions. Means, diamonds; box plot shows 5%-Q1-median-Q3-95% intervals. (**b**) Similarly, single-virion CHK-152 counts were determined at pH 7.4 and are shown in a histogram. Particles falling within the fitted Gaussian were selected for further analysis: 75% of the data points with a CHK-152 count of up to 90 per virion. * *p* < 0.05. Supplement to Figure 4.

**Figure A5:**
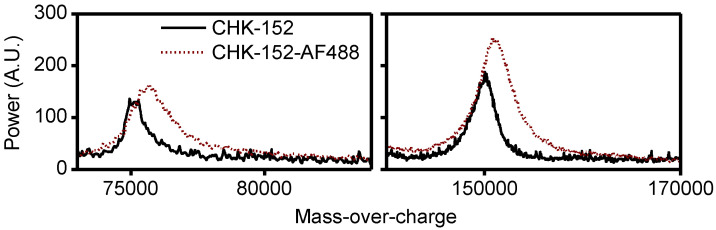
MALDI spectra of labeled and unlabeled CHK-152. Spectra were obtained with antibody dialyzed to 150 mM ammonium acetate. The AlexaFluor488 dye had a molecular weight of about 700. Supplement to Figure 5.

**Figure A6:**
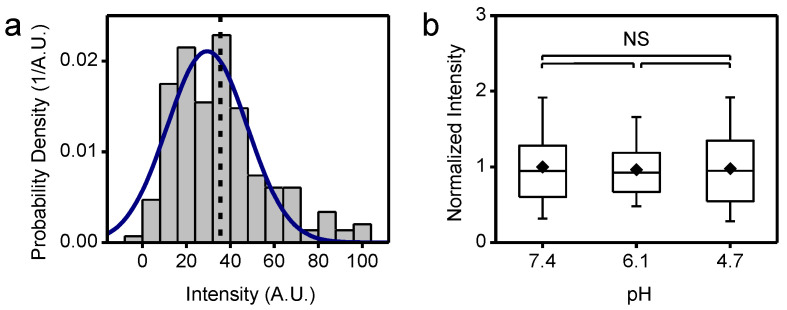
Labeled CHK-152 intensity determination; (**a**) single AF488-labeled CHK-152 were flown into the flow cell and absorbed aspecifically to the cover glass at pH 7.4. Imaging conditions as for a fusion experiment were then used to extract the single CHK-152-AF488 intensity. The histogram of intensities is shown for *n* = 186 spots. Solid line is a Gaussian fit, dashed line shows mean value; (**b**) with conditions as in panel a, the intensities of CHK-152-AF488 versus pH are shown, normalized to mean pH 7.4 intensity. Significances from *t*-test, *n* = 65, 47, 58 spots, respectively. Means, diamonds; box plot shows 5%-Q1-median-Q3-95% intervals. Supplement to Figure 5.

CHK-152 was labeled with AlexaFluor488 to enable quantification of the copy number bound to single virions. To this end, both the intensity of single, tagged CHK-152 and the unlabeled fraction of the antibody were determined (Methods). Because CHK-152 incubation induced some amount of virion aggregation, we analyzed 75% of the virus particles, those with the lowest antibody counts (more details in Methods). CHIKV was incubated with 0.63 nM of tagged CHK-152 for 15 min at 37 °C to allow binding to occur. This concentration resulted in an average of 52 ± 3 antibodies bound per virion with minor preparational variation per pH condition (Figure A3a). This number corresponds to 22–43% of the 240 epitopes bound, depending on the valency of CHK-152 binding (see Discussion). Under all conditions, this number of bound CHK-152 reduced the total extent of fusion (Figure 4a), indicating that CHK-152 directly blocks fusion at concentrations leading to submaximum epitope occupancy. The largest relative inhibition was observed at the threshold pH of 6.1 and 6.2. In addition to a reduction in extent, fusion was slowed down significantly under all pH conditions (as tested on the medians, Figure 4b). There was no consistent correlation between the fusion of particles and the starting antibody count (Figure A3b). This observation may indicate that only a small number of the CHK-152 bound determine the fate of fusion, a number small enough that it does not contribute a detectable correlation.

### 3.5. CHK-152 Dissociates from Viral Particles at Low pH

**Figure 5:**
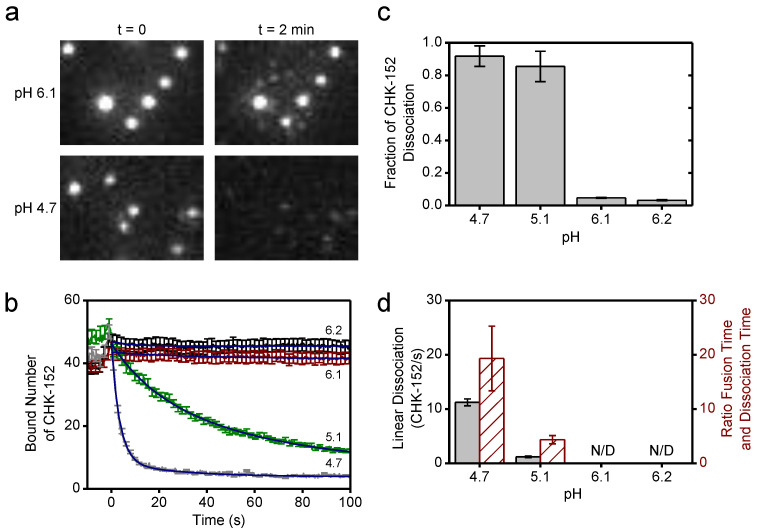
CHK-152 dissociation at low pH; (**a**) fluorescent spots of CHK-152 bound to virions are shown from a region of a movie slice for pH 6.1 and 4.7, and for *t* = 0 and *t* = 2 min. At pH 4.7, loss of CHK-152 from the virions was observed after 2 min. Image heights correspond to 8.5 μm; (**b**) the average of bound CHK-152 of nonfusing virions is shown over time. Increase of signal towards *t* = 0 was due to rolling and arrest of virus particles. One out of every five error bars shown, to reduce visual clutter. Blue lines show exponential fits (see text); (**c**) the final fraction of antibody remaining for each pH point was determined from the fits in panel b; (**d**) the linear rate of dissociation at *t* = 0 determined from the fits in panel b is shown per pH point in black (left *y*-axis). Red bars (right *y*-axis) show the ratio of the mean fusion time without antibody (see Figure 4) to the dissociation time (the inverse of the linear dissociation rate), at the pH points indicated. All error bars, sem. N/D: not detectable. Confirmation of CHK-152 labeling in Figure A5. Single CHK-152 intensity determination in Figure A6 and Methods. The average of bound CHK-152 of fusing virions is shown in Figure A7.

We observed that at pH 4.7 and 5.1, the fusion inhibition was reduced compared to the pH 6.1 and 6.2 conditions, even though the initial binding levels of CHK-152 were similar (Figure A3a). Hence, we decided to check the amount of CHK-152 bound to the virus particles over time. Figure 5a shows observed spots from single virions bound with fluorescently tagged CHK-152. After 2 min at pH 4.7, almost all fluorescence had disappeared from spots of nonfusing virions, indicating CHK-152 dissociation. In contrast, at pH 6.1, only marginal reduction of fluorescence was observed.

**Figure A7:**
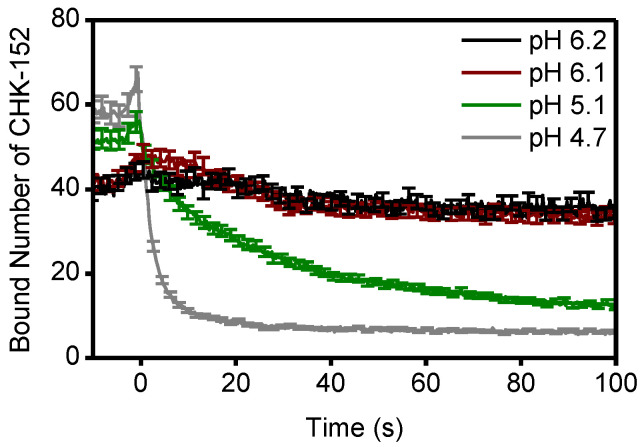
Bound number of CHK-152 averaged for all fusing particles over time. In the single-particle assay, the fluorescence intensity of virions was tracked over time and converted to absolute number of CHK-152 bound (Methods). The average number of CHK-152 bound for fusing virions is shown over time. Increase of signal towards *t* = 0 due to rolling and arrest of virus particles. One out of every five error bars (sem) shown to reduce visual clutter. Supplement to Figure 5.

The average bound number of CHK-152 over time was determined separately for fusing and nonfusing particles (Figure 5b and Figure A7). Time *t* = 0 was defined by the loss of fluorescence of the pH-sensitive fluorescein, and signals showed an initial increase towards *t* = 0 due to the rolling and arrest of virions under the force of the inflowing low-pH buffer. Both fusing and nonfusing virions displayed CHK-152 dissociation at pH 5.1 and 4.7. Because fusing particles additionally lost CHK-152 after fusion due to diffusion (Figure 5a, top images), we decided to take the number of CHK-152 bound to the nonfusing particles (Figure 5b) as a proxy for the dissociation behavior of the whole population, as this indicates purely dissociation into solution.

As the fusion yields were slightly different for pH 5.1 and 4.7, we determined the properties of CHK-152 dissociation for both pH points. The curves showing the number of bound CHK-152 over time were fit with single-exponential (pH 6.2 and 6.1) and double exponential (pH 5.1 and 4.7) decay functions to extract the fraction of CHK-152 that ultimately dissociated (Figure 5c). Only marginal loss of the antibody was observed at pH 6.2 and 6.1, whereas more than 80% of antibodies dissociated at pH 5.1 and 4.7. From the fits, the linear rate of dissociation at *t* = 0 was determined for pH 5.1 and 4.7 (Figure 5d, red), showing that pH 4.7 features an about 10-fold faster initial dissociation rate. Importantly, the ratio of the rates of fusion and rates of dissociation differed (Figure 5d, green): at pH 4.7, CHK-152 dissociation is about 10-fold faster than at pH 5.1, while the mean fusion time is only about 2-fold faster. The rate of dissociation may, therefore, explain the differences in extent of fusion at pH 5.1 and 4.7. We postulate that fusion would be blocked with the starting CHK-152 counts (such as at pH 6.1 and 6.2). However, due to sufficiently fast dissociation, compared to the timescale of the events leading to fusion and E1 protein inactivation, some virions would become fusogenic again. Dissociation happens more quickly at pH 4.7 than at 5.1, relative to the events that lead to membrane fusion, thereby leading to a higher fusion extent. We, therefore, numerically modeled the process leading to the observed fusion extents, taking the CHK-152 stoichiometry and dissociation into account.

### 3.6. Antibody Stoichiometry Indicates High Cooperativity at the Level of E1/E2 Spikes

The binding of CHK-152 blocked and slowed down fusion. However, most epitopes were not bound with CHK-152, and at pH 4.7, CHK-152 dissociated very quickly. To explain how small numbers of antibody can inhibit fusion, we devised a numerical model of fusion in which a single CHK-152 bound to an E2 surface epitope prevents the whole spike from participating in fusion. This model bears a resemblance to earlier work by us and others on influenza fusion inhibition [29,43]. Moreover, dissociation of all CHK-152 bound to the spike would restore that spike’s fusogenicity, provided the dissociation happened rapidly enough, compared to the fusion timescale. The fusion extent was then numerically evaluated by assessing the availability of a sufficient number of unbound spikes that are in contact with the target membrane. Comparison of the results of this model to the observed stoichiometries and dissociation properties can then inform us of the cooperativity of CHIKV fusion at the spike level.

**Figure 6:**
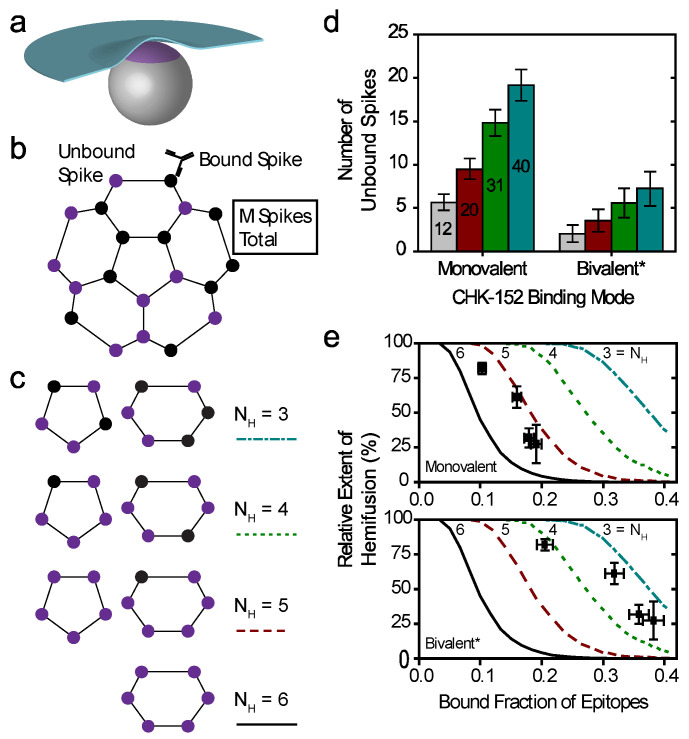
Cooperative model of CHIKV fusion at the level of spikes; (**a**) a virion (grey) docked to the planar membrane (blue) is shown. The region in contact with the target membrane is shown in purple: the contact patch; (**b**) the contact patch consists of *M* spikes, example of *M* = 20 shown. More details of the patch size are in Figure A8. Unbound spikes (purple) are considered to mediate fusion whereas spikes bound with one or more CHK-152 are considered not to (black); (**c**) cooperative fusion was modeled by the availability of a minimum number of unbound spikes, *N*_H_, in any of the 5- and 6-rings on the viral surface. The unbound positions can be anywhere in the ring; examples for different *N*_H_ are shown; (**d**) for 10,000 virions 52 ± 3 CHK-152 were randomly bound per virion. Both the contact patch was varied (from 12 to 40) and the CHK-152 binding mode. The mean ± SE of the number of unbound spikes is shown. Bivalent * binding was modeled as binding by 104 ± 6 monovalent Fabs; (**e**) for 10 000 virions CHK-152 was randomly bound as in panel d, and the relative extent of fusion was determined as the fraction of virions having available *N*_H_ free spikes in a ring, as defined in panel c. The extents of fusion from the simulations are shown as lines versus the fraction of CHK-152-bound epitopes on the viral surface. Line legends are as shown in panel c: *N*_H_ = 3, 4, 5, 6 are indicated by dash-dotted, dotted, dashed, and a solid line respectively. The experimental extent of fusion was determined relative to the no-antibody control (Figure A9) and is plotted versus the time-averaged fraction of bound epitopes (black squares, mean ± sem). This time average takes into account CHK-152 dissociation (see text and Figure A10). For panel e, different patch sizes and their influence on the best fit parameters is shown in Figure A11 and Figure A12.

The key parameters in the model were the total number of spikes associated with the target membrane and the number of spikes that need to cooperatively act to mediate fusion. We considered different sizes for the contact patch in interaction with the target membrane, containing *M* proteins (Figure 6a). A spike was considered not to participate in mediating fusion if one or more of the three spike epitopes were bound by antibody (Figure 6b). Fusion could only be attained if a virus particle had a number *N*_H_ of unbound spikes within any 5- or 6-ring in its contact patch. Here, *N*_H_ = 1 signifies fusion mediated by a single E1 trimer formed from an unbound spike, and for higher *N*_H_ fusion results from multiple unbound spikes in a ring on the viral surface (illustrated in Figure 6c). The positions of unbound spikes within the ring did not matter, as long as any ring in the contact patch contained *N*_H_ unbound spikes.

We considered the two extreme cases of the CHK-152 binding mode with its two Fab domains: pure monovalent and pure bivalent binding. With a number of 52 ± 3 antibodies bound over the 240 epitopes (in 80 spikes), the probability of a spike to be unbound is: *p_unboundSpike_* = (1–52/240)^3^ = 0.48 ± 0.03 for monovalent binding, or *p_unboundSpike_* = (1–104/240)^3^ = 0.18 ± 0.03 for bivalent * binding. We write bivalent * binding, as this was estimated as the binding of double the amount of monovalent Fabs. This is an unattainable maximum epitope occupancy, since bivalent antibodies can only bind neighboring epitopes, and additionally, will experience steric hindrance. Considering the probabilities calculated above, any contact patch of size *M* > 5, corresponding to greater than 6.25% of the virion surface, on average has more than one unbound spike in contact with the target membrane.

**Figure A8:**
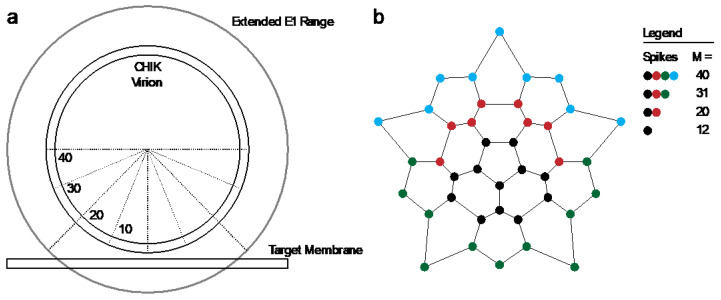
Patch size considerations; (**a**) schematic diagram of the number of spikes that fall within range of the contact patch (delineated by dotted lines) facing the target membrane. Virion of 65 nm diameter and E1 proteins of 13 nm length assumed (approximate range shown in grey). The number of spikes is shown, which fits on the relative fraction of the viral surface indicated. In total, the virion comprises 80 spikes; (**b**) layout of the surface grid of spikes of one half of a CHIK virion. The lines indicate the connections that make rings. The different contact patch sizes are indicated by color, cumulatively: *M* = 12 (black), *M* = 20 (black + red), *M* = 31 (black + red + green), *M* = 40 (black + red + green + blue). Supplement to Figure 6.

For a virion of 65 nm in diameter, we estimate the contact patch to be 20 spikes, or 25% of the viral surface by looking at the range that the 13 nm E1 [6] may reach to a planar target membrane (Figure A8a). Earlier work has similarly estimated the contact patch area of spherical, 50 nm diameter influenza viruses at 25% of the outer surface [44]. Here, the contact patch could be larger if inserting E1 were to pull the target membrane around the virion as a coat, or could be smaller due to steric hindrance of antibodies. In the biological context, the contact area with the inversely curved endosome may increase the contact patch. Therefore, we consider different sizes of *M* from 12 (about one eighth) to 40 (one half of a virion), as shown in Figure A8b, which appear to be reasonable limits for the minimum and maximum contact patch sizes, respectively. Then, we counted the number of unbound spikes in numerical simulations of the fusion. All tested patch sizes were determined to have multiple unbound spikes available on average (Figure 6d), in line with what we calculated above. We, therefore, considered a cooperative fusion mechanism.

**Figure A9:**
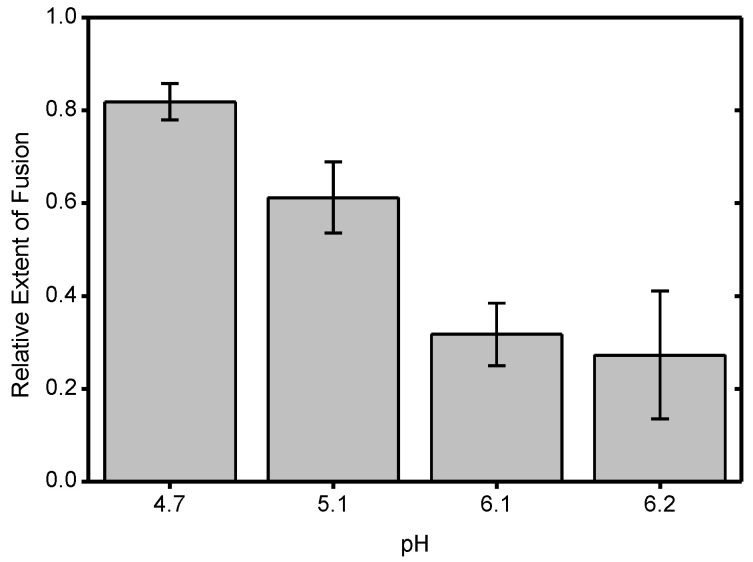
Relative extent of fusion with CHK-152 for each pH point. The relative extent of fusion was calculated as the ratio of the extents of fusion of the antibody and no-antibody conditions (both from Figure 4). SEM was propagated accordingly. Supplement to Figure 6.

**Figure A10:**
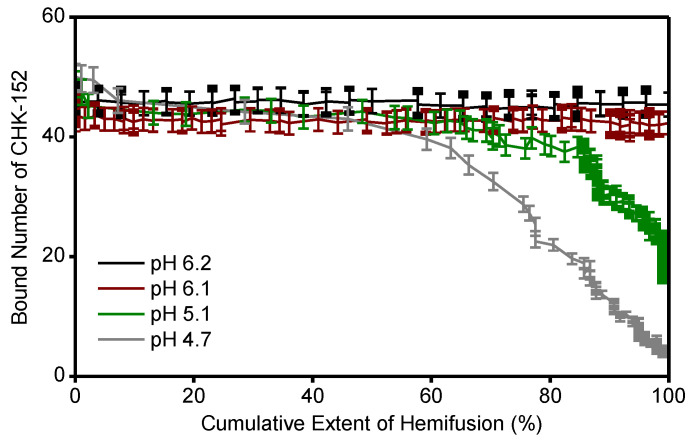
Correlation of the mean number of bound CHK-152 versus the cumulative extent of fusion. Both the extent of fusion and number of CHK-152 were determined over time for individual virions and then averaged. The two readouts are here plotted against each other for each time point. As is visible in Figure 5, at pH 6.2 and 6.1, only a small number of CHK-152 dissociate, whereas at pH 5.1 and 4.7 dissociation occurs. The graph shows that at pH 5.1 and 4.7, only late-fusing virions, with respect to the whole fusing population, lost large numbers of CHK-152 at the moment of fusion. Supplement to Figure 6.

First, we scaled the data to enable comparison with the numerical model. The extents of fusion in the presence of CHK-152 were calculated relative to the no-antibody condition, thereby correcting the extents for nonfusogenic virions and for the effect of pH on the total extent (Figure A9). To correct for the dissociation of CHK-152 over time, we then calculated the effective number of CHK-152 bound to the virus particles during the time they fuse. We calculated this effective number over the timescale of fusion, by averaging the number of CHK-152 bound to nonfusing virions over the population, and subsequently averaging over time weighted by the number of particles that had not yet fused (see Figure A10). It is, therefore, an estimate of the average number of CHK-152 a fusing virion had bound during the time to fusion. The result is shown in Figure 6e (squares): the observed relative extents of fusion versus the estimated effective epitope occupancies in the cases of monovalent and bivalent * binding.

**Figure A11:**
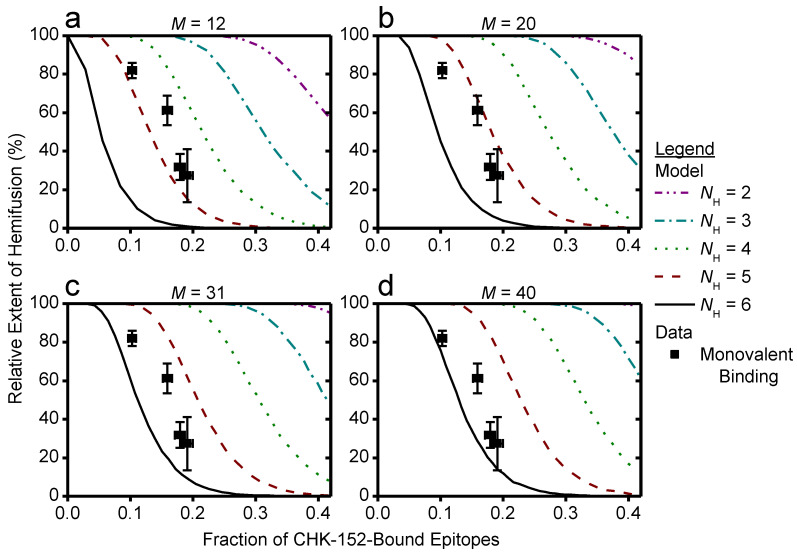
Simulation and data compared for different patch sizes, assuming monovalent CHK-152 binding. As in Figure 6, for 10,000 virions, CHK-152 was randomly bound and the relative extent of fusion was determined as the fraction of virions having available *N*_H_ free spikes in a ring. The extents of fusion from the simulations are shown as lines versus the fraction of CHK-152-bound epitopes on the viral surface. Line legends are as shown in Figure 6c: *N*_H_ = 3, 4, 5, 6 are indicated by dash-dotted, dotted, dashed, and a solid line, respectively. The data points (squares) shown are the same for every graph and are equal to that of Figure 6e. The simulation was adapted to assume a contact patch of *M* = 12 (**a**), 20 (**b**), 31 (**c**), 40 spikes (**d**) as indicated above the graphs. Supplement to Figure 6.

**Figure A12:**
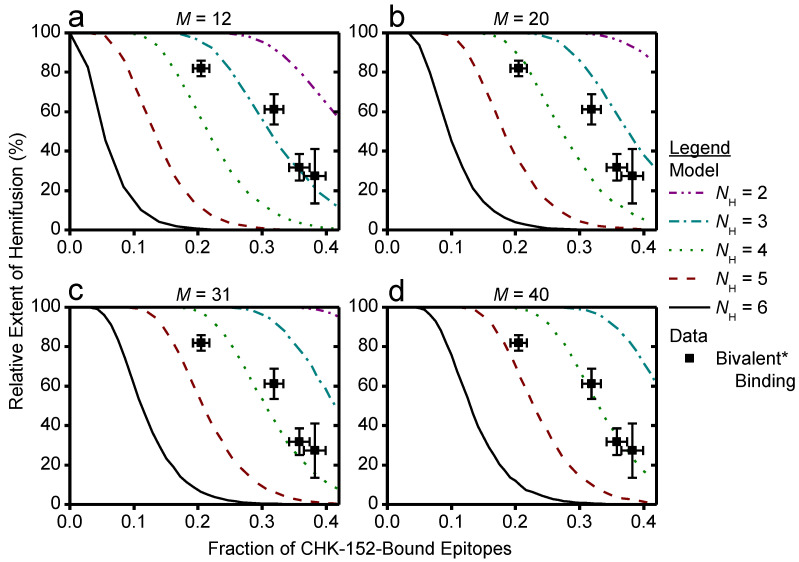
Simulation and data compared for different patch sizes, assuming bivalent CHK-152 binding. Simulation, legend, and data as for Figure A11, but assuming bivalent * binding of CHK-152. This was modeled by binding double the amount of Fabs. The simulation was adapted to assume a contact patch of *M* = 12 (**a**), 20 (**b**), 31 (**c**), 40 spikes (**d**) as indicated above the graphs. Rest of legend as in Figure A11. Supplement to Figure 6.

**Figure A14:**
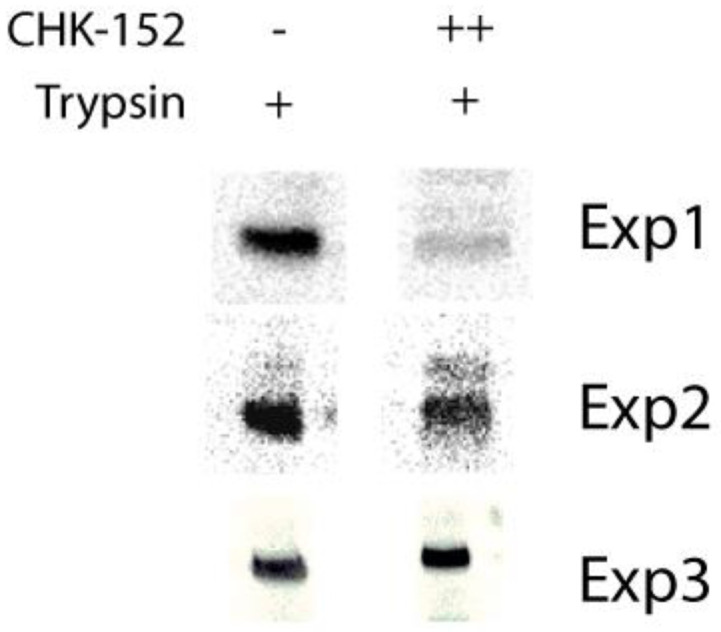
Triplicate data supplementary to Figure 2b: inhibition of formation of trypsin-resistant E1 trimer. Radiolabeled CHIKV was incubated with or without CHK-152 for 10 min at 37 °C, added to liposomes and acidified to pH 5.1. After 1 min, the sample was neutralized to pH 8.0. The sample was incubated with 0.25% β-ME for 30 min at 37 °C, trypsinized for 1 h and subjected to SDS-PAGE analysis. CHK-152 concentration at incubation: ++, 20 nM CHK-152 in ratio of 335 to virions.

Finally, we ran numerical simulations for 10,000 virions determining at each epitope occupancy what fraction of the virions had a ring containing *N*_H_ unbound spikes, defining the extent of fusion. The result is shown in Figure 6e as lines, for *M* = 20. We see that the data best match fusion mediated by 3–5 unbound spikes in a ring (indicated by a red dashed and cyan dash-dotted line, respectively), depending on the CHK-152 binding valency. The cooperativity was largely determined by the valency of CHK-152 binding; the actual contact patch simulated was of minor effect (Figure A11 and Figure A12).

## 4. Discussion

Here, we reported on the mechanism of action of the antibody CHK-152. We determined that it shields the virions at high concentrations of binding, preventing membrane interaction under neutral-pH as well as low-pH conditions. Using a single-particle fluorescence assay and a substoichiometric ratio of CHK-152 binding, virions were pre-docked to a membrane. This approach allowed us to determine that CHK-152 also plays a role in directly blocking the fusion step. In this assay, CHK-152 was observed to dissociate at low pH, whereas it remained bound at mildly acidic pH. We devised a numerical model of CHIKV fusion with only E1 from unbound spikes able to trimerize and mediate fusion, and in which fusion is achieved by insertion of a minimal number of E1 trimers within a ring of neighboring spikes. Correcting for CHK-152 dissociation, the CHK-152 stoichiometries of binding were not consistent with fusion by single E1 trimers, but rather with fusion mediated by three to five trimers.

In addition to CHK-152 effectively preventing viral docking to membranes at a neutral pH, it appears to directly block low-pH fusion by interfering with the stable attachment of the virus to the target membrane. Our data and previous work indicate that prevention of virus attachment to the cell, possibly by sterically hindering receptor or membrane interaction, is an important mechanism in its neutralizing efficiency [21]. We demonstrated that CHK-152 also directly inhibits fusion for predocked virions, at subsaturated occupancy of binding. This enhances its potential as an antiviral by the multiplicative effect of binding reduction and fusion inhibition. It has been shown previously that the CHK-152 Fab binds residues in the E2 A domain and the β-ribbon. The latter lies in the acid-sensitive region that becomes disordered at low pH, facilitating exposure of the E1 fusion loop [6,23,24]. As we find that CHK-152 prevents the formation of a trypsin-resistant form of E1, and inhibits stable association of E1 with target membranes, it seems plausible that CHK-152 inhibits E1 membrane insertion by blocking E1/E2 heterodimer dissociation. However, it could also lock the E2 proteins in place, allowing E1 membrane insertion but preventing trimerization, as observed in studies at the threshold pH of 6.4 for the Sindbis virus [17]. Interestingly, the acid-sensitive region and A and B domains appeared more often as binding targets for antibodies [37,39,40]. The epitope of neutralization lies within one single E2 molecule, in contrast with other, E2-crosslinking antibodies isolated for alpha- and flaviviruses [36,37,38], so ‘locking’ the virion would require CHK-152 bivalent binding.

We observed CHK-152 dissociation at pH 5.1 and 4.7. In the in vitro conditions of our experiment, all unbound CHK-152 had been washed away so that CHK-152 dissociating after acidification effectively disappeared. This is in contrast with the liposomal fusion conditions [21] and an in vivo situation, where CHK-152 might rebind from solution. Moreover, at the probed stoichiometry of binding in the single-particle assay, dissociation of just a couple of CHK-152 may restore virion fusogenicity. This would not be the case for higher concentrations of antibody incubation. Dissociation was marginal at pH 6.1 and 6.2, the pH of the early endosome through which CHIKV enters cells [13], and the extent of fusion was strongly reduced at these pH points. Moreover, the CHIKV strains so far have a sharp pH threshold and appear to be liable to acid-induced inactivation [14]. In all, CHK-152 dissociation may not need to compromise its neutralization effectiveness in vivo even at substoichiometric binding levels.

We found that the relative rate of CHK-152 dissociation determined the final extent of fusion for pH 4.7 and 5.1. However, at both pH points, nearly all CHK-152 dissociated if given enough time. Together, this indicates that there is a “window of opportunity” during which the spikes must become unbound in order to still be able to mediate fusion again. Such a window of opportunity may arise, for example, due to inactivation of E1 proteins at low pH, as observed without the presence of target membranes [14]. Even though the window of opportunity is an underlying, necessary assumption of our model, we did not explicitly model it as we just considered the average presence of CHK-152 for particles during the time they take to fuse.

Two different mechanisms of CHK-152 dissociation could be involved. In the first, the CHK-152 lose affinity due to protonation changes in the epitope or paratope. This may involve an antibody-induced shift of the p*K*_a_ of protonatable residues on the protein, as suggested in Zeng et al. [45]. In the second, we see an analogue to how the influenza hemagglutinin has been modeled to overcome the kinetic barrier to rearrange to the post-fusion state by protonation [46]. Here, the CHK-152 would raise the kinetic barrier for E2-E1 heterodimer dissociation. However, this increased barrier to conformationally rearrange is then overcome at sufficiently low pH, shedding the antibody. Identifying the dissociation mechanism is beyond this study, as both described changes in CHK-152 and viral protein are proton-triggered. However, it appears important to determine if this mechanism is common in antibody-mediated neutralization of class II viruses, if it allows decreased-pH-threshold escape mutants to arise and if this could be avoided or exploited in a rational antiviral design.

Employing the fusion-inhibiting capacity of CHK-152, we found CHIKV fusion to be cooperative by determining the stoichiometry of binding of CHK-152 and numerically simulating the resulting availability of CHK-152-free spikes on the virion surface. Fusion ensued when a sufficient number of unbound spikes were available to trimerize and together overcome the membrane fusion barriers. In this scenario, the E1 trimer fusion loops could associate to facilitate dimpling of both membranes, as detected before for the E1 ectodomain [19,47]. The proposed mechanism is analogous to that developed for influenza viral fusion, where multiple protein trimers need to mediate fusion, and the network of potentially cooperating trimers is disrupted by inhibitor binding [29,43]. Interestingly, in those studies, binding of an estimated quarter of epitopes resulted in significant fusion inhibition, similar to the occupancy probed here.

The combination of data and the numerical model allowed us to determine that CHIKV fusion is cooperative, but some uncertainties remain. To develop a more complete understanding of CHIKV fusion, it is necessary to probe a large range of inhibitor-binding concentrations and to obtain sufficient statistics to allow inference on the individual protein events to membrane fusion (for instance, the steps of heterodimer dissociation and E1 membrane insertion). The distribution of fusion times then allows inference on the underlying rate-determining steps [43,48,49]. Here, we were limited to substoichiometric levels of binding as CHK-152 prevented nonspecific membrane docking at high binding levels, and the statistics were too limited to determine the fusion time distributions. The actual number of E1 trimers involved in fusion depended, for the most part, on the valency of the CHK-152 and less on the size of the contact patch. We point out two additional factors why CHIKV fusion is more cooperative than we could probe. First, the CHK-152 inhibited nonspecific docking, and the virions may, therefore, have preferentially bound with a relatively sparsely CHK-152-covered section of the viral surface. The epitope occupancy in the contact patch is then relatively lower than on the rest of the particle, which implies a more cooperative fusion mechanism. Second, we see no reason a priori why E1 from different spikes would be prevented from forming a trimer together. Compared to our model, this would further increase the number of E1 trimers that could form in the contact patch, thereby also implying a more cooperative mechanism.

Because of the reasons stated above, future studies should uncouple the binding- and fusion-inhibiting action of inhibitors by artificially coupling viruses to the membrane surface. Furthermore, using monovalent-binding Fab fragments eases interpretation of the data, and may reduce steric effects. Our results on alphavirus fusion fit in with a universal context found so far across all three classes of enveloped viruses, where fusion is mediated by multiple protein trimers in a close neighborhood [44,48,49]. Taken together, our data identifies important parameters to consider in the rational development of CHIKV antivirals.

## Figures and Tables

**Figure A13:**
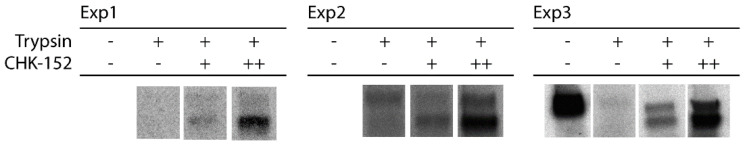
Triplicate data supplementary to Figure 1b: shielding of surface proteins from enzymatic cleavage. The [35S]-methionine/L-[35S] cysteine-labeled CHIKV was incubated with CHK-152 and mixed with liposomes at neutral pH. The mixture was trypsinized for 1 h and subjected to SDS-PAGE analysis. CHK-152 concentration in final volume: +, 0.63 nM CHK-152 in estimated ratio of 13 to virions; ++, 10 nM CHK-152 in ratio of 210 to virions.

## Data Availability

All data in graphs and statistical analyses used in this study are included as supplemental information in Appendix A, as well as representative, compressed examples of the fusion movies. Raw data, consisting of the fusion image captures, is available upon request due to their size of over a GB per file.

## Supplementary Materials

The following are available online at https://www.mdpi.com/article/10.3390/v14020270/s1, Video S1: CHIKV fusion movie at pH 4.7 without antibody CHK-152, Video S2: CHIKV fusion movie at pH 4.7 with antibody, Video S3: CHIKV fusion movie at pH 5.1 without antibody, Video S4: CHIKV fusion movie at pH 5.1 with antibody, Video S5: CHIKV fusion movie at pH 6.1 without antibody, Video S6: CHIKV fusion movie at pH 6.1 with antibody, Video S7: CHIKV fusion movie at pH 6.2 without antibody, Video S8: CHIKV fusion movie at pH 6.2 with antibody.

## Appendix A

**Table A1:** Significance testing. Supplement to Figure 4.

**A. Yield of Fusion (Figure 4a)**				
Observable		Yield of Hemifusion		
Compared conditions		No antibody (1) vs. 0.63 nM CHK-152 (2)		
Null hypothesis		Equal means		
Hypothesis test		Two-sided weighted Student’s *t*-test		
pH	Weights1 (number of particles)	Weights2 (number of particles)	*p*-value	
4.7	83, 44, 54	113, 10, 12	0.001	
5.1	149, 248, 142	25, 20, 113	5 × 10^−4^	
6.1	254, 227, 202	164, 13, 13, 19, 11	2 × 10^−5^	
6.2	249, 227, 270	105, 185, 10	0.01	
**B. Time of fusion (Figure 4b)**				
Observable		Fusion time		
Compared conditions		No antibody (1) vs. 0.63 nM CHK-152 (2)		
Null hypothesis		Equal medians		
Hypothesis test		Two-sided Wilcoxon rank sum ^1^		
pH	n1	n2	*p*-value	
4.7	163	98	0.002	
5.1	490	91	2 × 10^−11^	
6.1	550	61	3 × 10^−11^	
6.2	188	26	0.003	

^1^ Reference: Nonparametric Hypothesis Testing: Rank and Permutation Methods with Applications in R, Bonnini et al.

**Table A2:** Fitting functions used and resulting fit parameters. Supplement to Figure 5.

Number of Bound CHK-152 Versus Time (Figure 5b)		
Fit Function: y = A1 *exp(−x/t1) + y0		
pH:	6.2	6.1
Parameter:		
Baseline y0	45.48 ± 0.03	41.45 ± 0.08
Amplitude A1	1.5 ± 0.1	2.05 ± 0.08
Time scale t1	8 ± 1	36 ± 4
**Fit function: y = A1 *exp(-x/t1) + A2 *exp(−x/t2) + y0**		
pH:	5.1	4.7
Parameter:		
Baseline y0	6.7 ± 0.2	3.83 ± 0.03
Amplitude A1	27 ± 2	4.9 ± 0.3
Time scale t1	59 ± 3	26 ± 2
Amplitude A2	12 ± 2	38 ± 2
Time scale t2	17 ± 2	3.4 ± 0.2
