# Supplementary material for: Cooperative Chikungunya Virus Membrane Fusion and Its Substoichiometric Inhibition by CHK-152 Antibody
Source: Viruses. 2022 Jan 28;14(2):270. doi: 10.3390/v14020270 (PMC8877538; doi:10.3390/v14020270)
Supplement: Supplementary file 1 [file viruses-14-00270-s001.zip › viruses-1512485-supplementary.pdf]

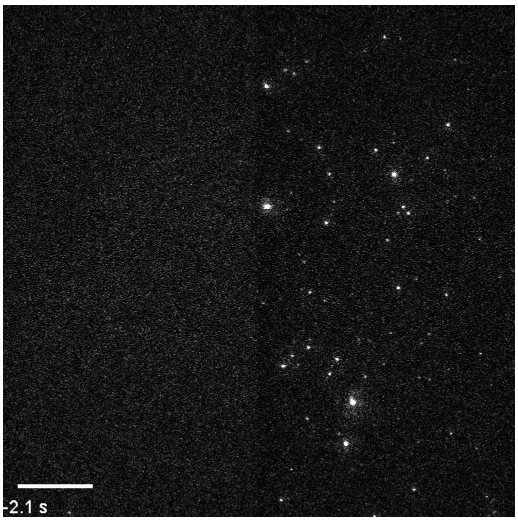

Video S1. Single-particle CHIKV fusion at pH 4.7 without CHK-152. Scale bar 20 µm. Realtime timelapse. For all videos, the following legend applies. Left-hand side, antibody fluorescence of virions docked to planar membrane. Right-hand side, fluorescence of virion membrane-located dye. At time $t = 0$ s, a faint dimming of the left-hand side background fluorescence indicates that the pH has dropped (as explained in more detail in Figure 3).

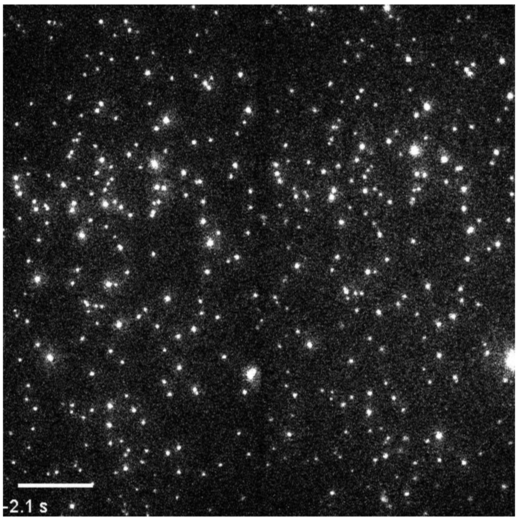

Video S2. Timelapse of single-particle CHIKV fusion at pH 4.7 with CHK-152. Scale bar 20 µm. Realtime timelapse.

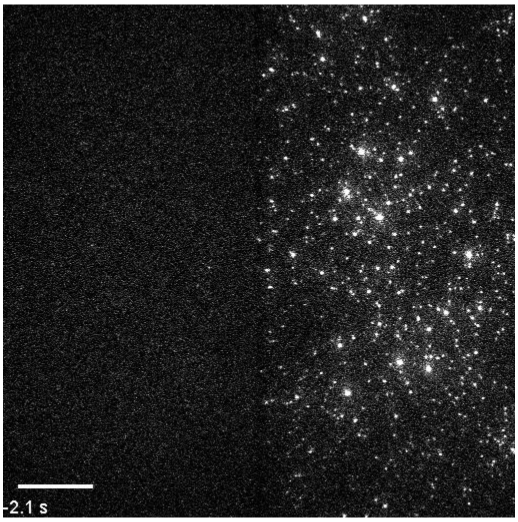

Video S3. Timelapse of single-particle CHIKV fusion at pH 5.1 without CHK-152. Scale bar 20 μm. Realtime timelapse.

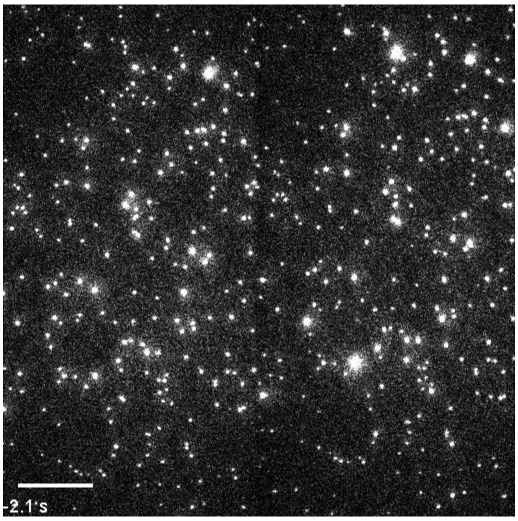

Video S4. Timelapse of single-particle CHIKV fusion at pH 5.1 with CHK-152. Scale bar 20 μm. Realtime timelapse.

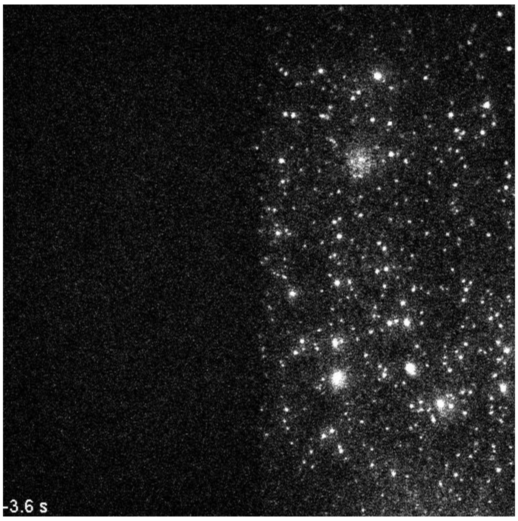

Video S5. Timelapse of single-particle CHIKV fusion at pH 6.1 without CHK-152. Scale bar 20 μm. Timelapse at 3× speed.

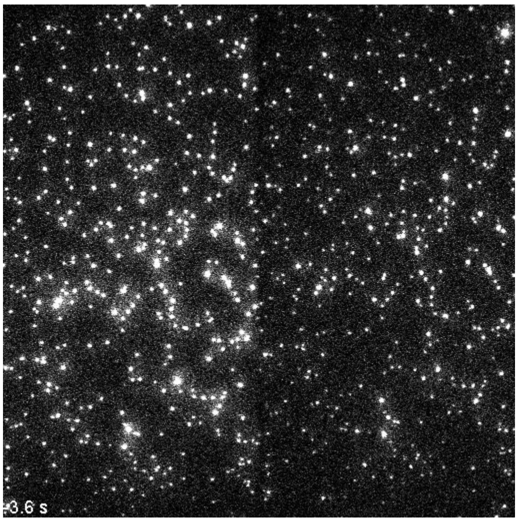

Video S6. Timelapse of single-particle CHIKV fusion at pH 6.1 with CHK-152. Scale bar 20 μm. Timelapse at 3× speed.

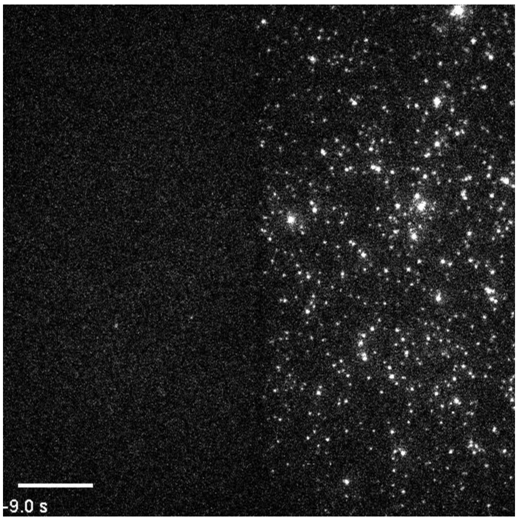

Video S7. Timelapse of single-particle CHIKV fusion at pH 6.2 without CHK-152. Scale bar 20 μm. Timelapse at 5× speed.

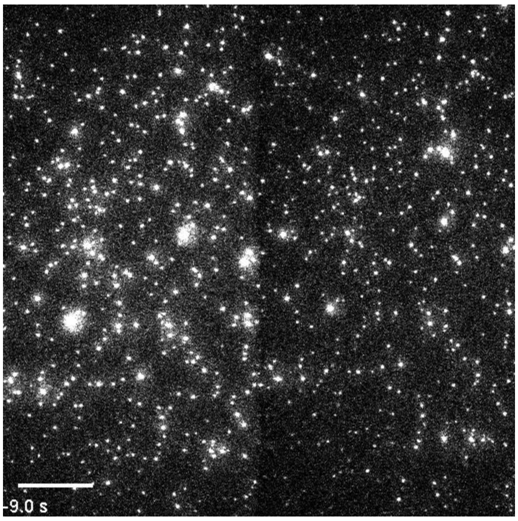

Video S8. Timelapse of single-particle CHIKV fusion at pH 6.2 with CHK-152. Scale bar 20 μm. Timelapse at 5× speed.
